# The Burden of COVID-19 in Children and Its Prevention by Vaccination: A Joint Statement of the Israeli Pediatric Association and the Israeli Society for Pediatric Infectious Diseases

**DOI:** 10.3390/vaccines10010081

**Published:** 2022-01-06

**Authors:** Michal Stein, Liat Ashkenazi-Hoffnung, David Greenberg, Ilan Dalal, Gilat Livni, Gil Chapnick, Chen Stein-Zamir, Shai Ashkenazi, Lior Hecht-Sagie, Zachi Grossman

**Affiliations:** 1 Infectious Diseases and Infection Control Unit, Hillel Yaffe Medical Center, Hadera 3810101, Israel; 2 Rappaport Faculty of Medicine, Technion—Israel Institute of Technology, Haifa 3109601, Israel; 3 Department of Day Care Hospitalization, Schneider Children’s Medical Center, Petah Tikva 4920235, Israel; LiatA3@clalit.org.il; 4 Sackler Faculty of Medicine, Tel Aviv University, Tel Aviv-Yafo 6997801, Israel; dalal@wmc.gov.il (I.D.); lgilat@clalit.org.il (G.L.); 5 The Pediatric Infectious Disease Unit, Soroka Medical Center, Be’er Sheva 8458900, Israel; dudi@bgu.ac.il; 6 The Faculty of Health Sciences, Joyce & Irving Goldman Medical School at Ben Gurion University of the Negev, Be’er Sheva 8410501, Israel; 7 Pediatric Department, E. Wolfson Medical Center, Holon 5822012, Israel; 8 Department of Pediatrics A, Schneider Children’s Medical Center, Petah Tikva 4920245, Israel; 9 Maccabi Healthcare Services, Tel Aviv-Yafo 6812509, Israel; chapnick@mac.org.il (G.C.); hecht_l@mac.org.il (L.H.-S.); zgrosman@netvision.net.il (Z.G.); 10 Jerusalem District Health Office, Jerusalem 9137001, Israel; chen.zamir@lbjr.health.gov.il; 11 Braun School of Public Health and Community Medicine, Faculty of Medicine, Hebrew University of Jerusalem, Jerusalem 9112102, Israel; 12 Schneider Children’s Medical Center, Petah Tikva 4920235, Israel; shaias@ariel.ac.il; 13 Adelson School of Medicine, Ariel University, Ari’el 4070000, Israel

**Keywords:** COVID-19, children, adolescents, vaccine, risk-benefit, efficacy, safety, transmission

## Abstract

As of October 2021, SARS-CoV-2 infections were reported among 512,613 children and adolescents in Israel (~33% of all COVID-19 cases). The 5–11-year age group accounted for about 43% (223,850) of affected children and adolescents. In light of the availability of the Pfizer-BioNTech BNT162b2 vaccine against COVID-19 for children aged 5–11 years, we aimed to write a position paper for pediatricians, policymakers and families regarding the clinical aspects of COVID-19 and the vaccination of children against COVID-19. The first objective of this review was to describe the diverse facets of the burden of COVID-19 in children, including the direct effects of hospitalization during the acute phase of the disease, multisystem inflammatory syndrome in children, long COVID and the indirect effects of social isolation and interruption in education. In addition, we aimed to provide an update regarding the efficacy and safety of childhood mRNA COVID-19 vaccination and to instill confidence in pediatricians regarding the benefits of vaccinating children against COVID-19. We reviewed up-to-date Israeli and international epidemiological data and literature regarding COVID-19 morbidity and its sequelae in children, vaccine efficacy in reducing COVID-19-related morbidity and SARS-CoV-2 transmission and vaccine safety data. We conducted a risk–benefit analysis regarding the vaccination of children and adolescents. We concluded that vaccines are safe and effective and are recommended for all children aged 5 to 11 years to protect them from COVID-19 and its complications and to reduce community transmissions. Based on these data, after weighing the benefits of vaccination versus the harm, the Israeli Ministry of Health decided to recommend vaccination for children aged 5–11 years.

## 1. The Burden of COVID-19

### 1.1. The Epidemiology of SARS-CoV-2 Infection in Children and Adolescents

As of late October 2021, the Israeli Ministry of Health (IMOH) reported 512,613 children and adolescents with proven severe acute respiratory syndrome coronavirus 2 (SARS-CoV-2) infection in Israel (~33% of all COVID-19 cases). The 5–11-year age group accounted for about 43% (223,850) of affected children and adolescents (unpublished data, personal communication). The relatively high incidence of this age group reflects an inverse relation to the immunization rate [1]. As the immunization coverage rates of adults and adolescents increased, the relative proportion of all new COVID-19 cases in these age groups decreased, whereas that of children, specifically of children aged 5–11 years, increased. Thus, as of December 2021, children and adolescents under the age of 18 years constituted more than 50% of the confirmed cases. IMoH data show that during the fourth wave (June–October 2021) of the COVID-19 pandemic, 3192 (0.6%) children and adolescents had recurrent positive PCR testing (unpublished data, personal communication). Since June 2021, the main circulating SARS-CoV-2 variant in Israel is the Delta variant (B.167.2); this variant of the fourth wave of the pandemic is nearly 50% more contagious than the Alpha variant (B.1.1.7), which dominated during the third wave of the pandemic [2]. The fourth wave emerged among 12-to-15-year-old adolescents [3]; this is consistent with the model by which the chance of transmitting new variants increases in parallel to a low immunization rate [4].

### 1.2. COVID-19 Morbidity and Mortality among Children and Adolescents, Including Risk Factors for Severe COVID-19 

Although COVID-19 morbidity and mortality are significantly lower in children than in adults, the risk of severe COVID-19 is not negligible, even among previously healthy children. A review of 1475 children from various countries who were hospitalized with acute COVID-19 reported a moderate to severe degree of illness in 615 (42%) [5]. In a prospective multicenter study in Israel [6], including 579 children hospitalized for COVID-19 or multisystem inflammatory syndrome in children (MIS-C), 103 had moderate to severe illness. Among them, 20% of those with COVID-19 and 56% of those with MIS-C were hospitalized in pediatric intensive care units, 7% and 20% of these respective groups necessitated mechanical ventilation.

Similar findings were described by the United States Center for Disease Control and Prevention (CDC) [7] regarding 204 hospitalized adolescents with COVID-19, about one-third of whom required hospitalization in intensive care units and 5% needed resuscitation. No mortalities were reported. 

Additionally, the CDC reported COVID-19 infection among 5,360,582 children aged 0–17 years in the United States from August 2020 to late October 2021 [7]. The number of COVID-19-related hospitalizations reported in this age group was 66,560. Of children aged 5–11 years hospitalized for COVID-19, about one-third were hospitalized in intensive care units [7]. 

The number of hospitalizations of children aged 0–17 years due to COVID-19 dramatically increased during the Delta wave of the pandemic. Reports from the United States showed a five-fold increase in hospitalizations [8]. However, the rate of severe disease among hospitalized children, as evident from admissions to intensive care units, remained constant. The rate of hospitalizations of unvaccinated adolescents was ten-fold higher than in fully vaccinated adolescents. Likewise, COVID-19-related hospitalizations were four-fold higher in countries with low COVID-19 immunization coverage compared with countries with high immunization coverage [9]. In countries with low COVID-19 immunization coverage, significant increases in emergency room visits were observed as well [8]. 

Despite the overall increase in the number of pediatric hospitalizations during the Delta wave, a decrease in hospitalizations among adolescents aged 16–18 years was witnessed in Israel compared to the third wave of the pandemic [3]. This reduction can be attributed to the beneficial effect of COVID-19 immunization in this age group. A decline in hospitalizations was also apparent for young children under age 4 years. A plausible explanation may be the close physical contact between this age group and their parents, which may have been favorably affected by the high COVID-19 immunization coverage in the adult population.

Reports from Israel indicate that as of late October 2021, 2660 children under age 18 years were hospitalized due to COVID-19 (unpublished data, personal communication), constituting approximately 0.5% of all SARS-CoV-2-positive children during this period. This is in accordance with the reported hospitalization rates of 0.1–2% described by the American Academy of Pediatrics [10]. Of the 2660 hospitalized children, 398 exhibited a moderate to critical acute illness, meaning that 1 in 7 children hospitalized for acute COVID-19 in Israel experienced a moderate to critical condition. Of these, 11 children died; two were newborns to COVID-19-ill mothers and nine had underlying illnesses. Of 460 hospitalized children aged 5–11 years, 72 were hospitalized in moderate-to-critical condition: three of them died (unpublished data, personal communication). These data indicate that the estimated risk for moderate/severe/critical acute COVID-19 is 1:1280 SARS-CoV-2-positive Israeli children, and 1:3000 among those aged 5–11 years.

Factors that have been associated with moderate-to-severe COVID-19 in children include underlying diseases, such as neurological diseases, congenital syndromes, obesity, diabetes, hematologic diseases, malignancies and immunodeficiency [5]. However, importantly, 61% of the children with moderate-to-severe COVID-19 were previously healthy. As for MIS-C syndrome, obesity was present in 13% of patients; however, the vast majority (83%) were previously healthy children. Of the 5–11 age group, 54% of children with moderate-to-severe COVID-19 and 88% with MIS-C were previously healthy (unpublished data, personal communication).

**Figure 1:**
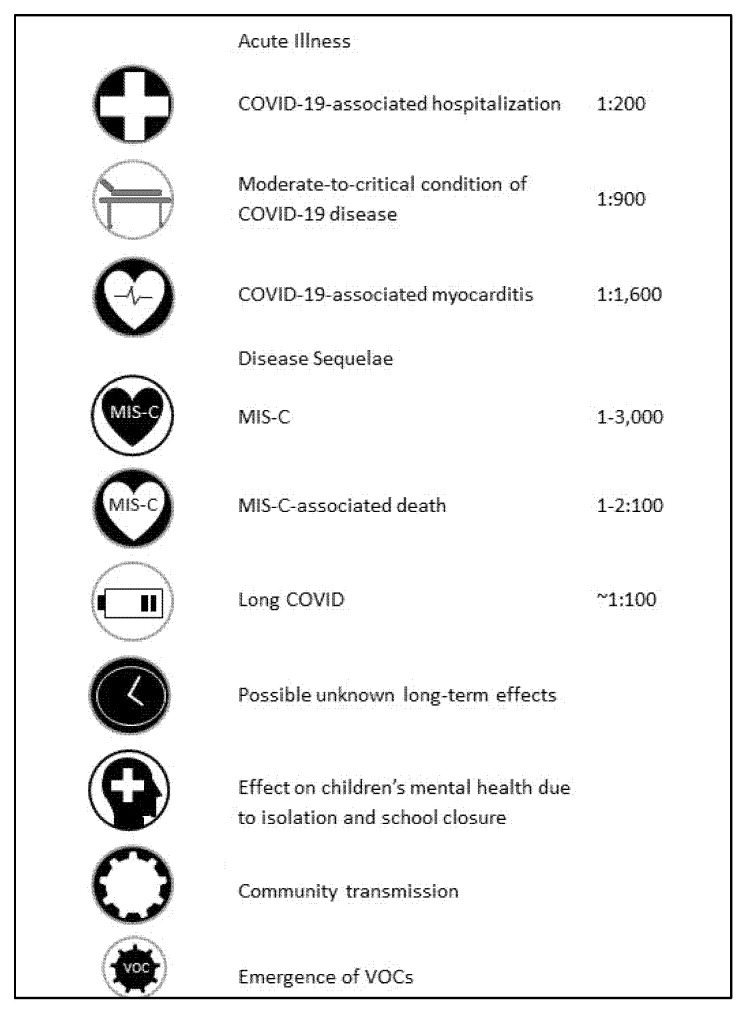
Summary of risks of COVID-19 disease in children.

A similar spectrum of background illnesses was described in the review mentioned by the CDC [7]. Of 204 hospitalized adolescents with COVID-19, 71% presented with obesity; chronic lung disease, including asthma; neurological disorders; metabolic diseases, including diabetes; immunodeficiency; hematological diseases; and cardiovascular diseases. Thirty percent did not exhibit any underlying illness, including obesity. Among children aged 5–11 years in the United States, significantly higher mortality rates occurred from COVID-19 than from vaccine-preventable diseases [9]. The risks of COVID-19 disease in children are summarized in Figure 1.

## 2. Long-Term Complications of COVID-19 Infection

### 2.1. Myocarditis as a Complication of COVID-19

An important aspect of the morbidity associated with acute COVID-19 infection is the complication of myocarditis. In a literature review of more than 52,000 COVID-19 confirmed individuals aged 19–74 years, about 30% experienced cardiac sequelae, mainly myocarditis in the short term and diastolic impairment at a later point [11]. Furthermore, follow-up of 1597 college student athletes who had been infected with COVID-19 demonstrated pathological findings on cardiac MRI in 2.3%. Clinically significant myocarditis was found in 0.5% (1:200) [8]. The CDC concluded that SARS-CoV-2 infection is associated with a substantially increased risk of myocarditis of 16-fold higher in the general population and 30-fold higher in children under age 16 years [9].

### 2.2. Multisystem Inflammatory Syndrome in Children (MIS-C)

MIS-C, also known as pediatric inflammatory multisystem syndrome, appears several weeks after SARS-CoV-2 infection. Manifesting as the multi-system involvement of at least four systems, the syndrome is characterized by prolonged fever, hypotension, gastrointestinal symptoms, rash, myocarditis and increased inflammatory indices. The proposed mechanism is post-viral immune dysregulation. Compared with acute COVID-19 disease in which pulmonary involvement is dominant, in MIS-C, the primary involvement is cardiac and includes multi-system inflammation [10,12]. The average age is 9 years and about 60% of those diagnosed are males. Worldwide reports [13,14,15] and those from Israel [6] indicate that the vast majority of patients with MIS-C do not have any background disease (including obesity).

According to the CDC [16], as of the beginning of October 2021, 5217 children in the United States were diagnosed with MIS-C, of whom, 46 have died. The average age was approximately 9 years. The age group of 5–11 years constituted 44% of the children. The majority of children (60–70%) were admitted to intensive care units. According to that report, the prevalence of MIS-C in the United States was approximately 1:3200 of SARS-CoV-2-positive children and adolescents, similar to the reported prevalence in Israel [6].

In a large series from the United States [17,18], 80% of children diagnosed with MIS-C were admitted to intensive care units, where about 53–80% had cardiac involvement and 20% needed mechanical ventilation. Mortality was 1–2%. An Israeli study showed similar findings: during the first three waves of the COVID-19 pandemic, 56% of children diagnosed with MIS-C were admitted to intensive care units and 20% required mechanical ventilation [6].

According to the IMoH, of 495,541 children under age 17 years with proven SARS-CoV-2, 221 were diagnosed with MIS-C (rate of 1:2240), of whom, one previously healthy child died (unpublished data, personal communication).

### 2.3. Long COVID in Children and Adolescents

As our understanding of the long-term consequences of COVID-19 advances, reliable data on long COVID or the post-COVID-19 condition in children and adolescents accumulate. The incidence of this syndrome varies, reaching up to 30%, depending on the population assessed, methods of data collection and the time elapsed from the acute illness [19,20,21,22,23,24,25,26,27,28]. Nevertheless, the clustering of symptoms and the results of a large-scale controlled study provide evidence of the long-term physical and mental illness in some children recovering from COVID-19 [19].

A study that assessed residual symptoms among 518 children hospitalized in Russia for COVID-19 found that 25% experienced symptoms several months after hospitalization [25]. However, lower rates were described by an online survey in the United Kingdom among 297,743 individuals over age 2 years based on self-reported or parental reports of long COVID. Four weeks after the acute illness, 0.2% of children aged 2–11 years and 0.9% of children aged 12-16 years had symptoms of long COVID [29]. A survey conducted by the IMoH reported residual symptoms 6 months after acute illness in between 1.8% and 4.6% of 13,834 SARS-CoV-2-positive children, depending on their age [30].

Research conducted in the United Kingdom as part of the CLoCk study compared symptoms between 3065 SARS-CoV-2-positive children, aged 11-17 years, and 3739 SARS-CoV-2-negative children. Three months post testing, SARS-CoV-2-positive children exhibited higher incidences of more than three (symptoms 30% vs. 16% in the control group) [21]. A large-scale study in Germany compared 96 health outcomes aggregated into 13 symptom complexes between 11,950 SARS-CoV-2-positive children and a control group [19]. Among the SARS-CoV-2 positive children, incidences of physical and mental illness were increased three months after the acute illness, including fatigue (incidence rate ratio (IRR) = 2.28, 95% CI = [1.71–3.06]), cough (IRR = 1.74, 95% CI = [1.48–2.04]) and chest pain (IRR = 1.72, 95% CI = [1.39–2.12]). Reported risk factors for long COVID in children included older age, female gender, atopic background, acute symptomatic disease and co-morbidities [19,24,25,27].

The range of symptoms observed in children includes fatigue, shortness of breath, cough, chest pain, headache, dizziness, muscle and joint pain, numbness, tremor, hair loss, difficulty sleeping and anxiety [24,26,28]. In a designated ambulatory clinic for long COVID in children at a tertiary center in Israel, 60% of the patients had significant disabilities in daily functioning four months after the acute illness, although most had only mild symptomatic acute illness. The mean patient age was 12 ± 5 years. Thirty percent were aged 5–11 years. Following a comprehensive medical evaluation, one-third of these children had abnormal respiratory findings, which were mostly compatible with a mild obstructive respiratory disease [28].

### 2.4. Possible Long-Term Sequelae of COVID-19

Beyond the ongoing complications currently known as long COVID, there is concern for future, yet unknown, long-term sequelae of COVID-19, as appears in other viral illnesses, such as measles, hepatitis B, hepatitis C, human papilloma virus, varicella and the Epstein–Barr virus [31]. A controlled study (prior to peer review) in the United Kingdom [32] presented findings indicative of brain injury in adult post-SARS-CoV-2 infection, irrespective of acute disease severity, with elements of gray matter depletion. The strengths of the study were its availability of imaging studies performed prior to SARS-CoV-2 infection and imaging studies of healthy controls. The researchers expressed concern that the changes observed may indicate future risk for various types of dementia. The pathogenesis for these findings might be explained by the finding of a unique injury of SARS-CoV-2 of cerebral blood vessels [33] using the viral protease, Mpro, which causes endothelial brain cell death. Another research raised the possibility of direct nerve damage as a basis for audio-vestibular injury following SARS-CoV-2 infection [34].

## 3. Infectivity and Transmission among Children and Adolescents

### 3.1. Infectivity of Children and Adolescents

Children and adolescents have similar or higher viral loads of SARS-CoV-2 compared to adults [35,36,37,38,39]. However, several studies globally [39,40,41,42] and in Israel [43] showed lower infectivity rates among children than adults. Infectivity rises with age, where adolescents present similar infectivity rates as adults [44,45,46,47,48,49].

In an analysis of COVID-19 infectivity according to age groups conducted by the Division of Epidemiology of the Public Health Services of the IMoH, infectivity of children changed throughout the waves of the COVID-19 pandemic. During the first and second waves between February 2020 and November 2020, only 23–32% of children and 15% of adults contracted COVID-19 infection from children [43]. In contrast, during the third wave, higher proportions of children and adults contracted the disease from children, with 40–51% and 29%, respectively. Data on the infectivity of children during the fourth wave (Delta wave) were retrieved from ~190,000 epidemiological investigations. Forty-nine percent of all infected persons were found to have contracted COVID-19 infection from children and adolescents aged ≤17 years and one-third from children aged 5–12 years (unpublished data, personal communication). Thus, with the availability of adult immunization, the role of children in the transmission of SARS-CoV-2 increased. Therefore, vaccination of children aged 5-11 years is anticipated to reduce the spread of SARS-CoV-2. A model proposed by the CDC [9] estimates that vaccination against COVID-19 in this age group will reduce the overall incidence of the infection by 8% in the forthcoming months.

### 3.2. The Role of Children in Household Transmission of COVID-19

Research on the infectivity of children within households has yielded mixed results. Several studies showed that transmission from children to household contacts was rare [50,51,52,53,54], while others report that transmission to household members was similar and even higher from children than from adults [39,49,55,56,57]. The variability in findings can be attributed to differences in community COVID-19 infection rates, methods of diagnosing secondary cases, the timing of sample collection and the level of adherence to infection control measures at home, which is particularly challenging when the index case is a young child [58,59].

### 3.3. The Role of Children in the Transmission of COVID-19 in Educational Institutions

Limited evidence suggests that COVID-19 infection is transmitted by adolescents within educational institutions and child care programs, and can be reduced by certain infection control practices, such as small class size, hygiene measures, masks and social distancing [39,47,60,61,62,63,64,65,66,67,68,69,70]. Of note, the physical conditions of the Israeli education system preclude the implementation of social distancing. 

## 4. Indirect Effects of COVID-19 on Children

### The Importance of School Routine

According to the Israeli Ministry of Education (personal communication, unpublished data), about one-third of the general pediatric population of Israel were under obligatory isolation due to COVID-19 infection or suspicion of infection. The mean number of days of isolation was 6.4–7.3.

The closure of educational institutions during the pandemic resulted in significant psychosocial damage to children and adolescents [71,72,73], including anxiety disorders, eating disorders, behavioral changes, decreased academic abilities and increased exposure to domestic violence. In surveys on social resilience conducted in Israel, 25.8% of responders noted worsening in their children’s mental health [74]. The Bank of Israel stated [75] that school closure may have long-term negative effects on the future income of children during their adult life and may increase social gaps. Therefore, continuing school routine and avoidance of isolation of children and adolescents are of paramount importance to their physical and emotional health.

## 5. Vaccine Efficacy 

### 5.1. The Efficacy of the Messenger Ribonucleic Acid (mRNA) COVID-19 Vaccine

The efficacy of one dose of the Pfizer-BioNTech BNT162b2 vaccine against symptomatic disease by the Delta strain was found to be relatively low at 33.5%; however, the efficacy following two vaccine doses increased to 87.9% [76]. This finding highlights the importance of a two-dose schedule of immunization for maximum protection.

Cumulative information regarding the effects of the vaccine in Israel on a population level shows that parallel to decreasing infection rates in adults, infection rates in children also decreased, despite the absence of vaccination in the latter. This pattern was observed in the third and fourth waves of the pandemic, in support of the contribution of indirect protection. Nevertheless, this indirect protection is insufficient in the context of a SARS-CoV-2 variant of higher infectivity in an unvaccinated population, as occurred during the Delta wave. 

### 5.2. The Efficacy of the mRNA COVID-19 Vaccine among Children Aged 5–11 Years 

Prior to the United States Food and Drug Administration (FDA) discussion to approve vaccination of children aged 5–11 years, which was held on 26 October 2021, data from the clinical study of the Pfizer-BioNTech BNT162b2 vaccine [77] were published. The vaccine is given at a dose of 10 micrograms, which is one-third the dose given to children aged ≥12 years, in a two-dose schedule with a 3-week interval between doses. The study included 1518 children who received the vaccine and 750 children who received a placebo. The level of neutralizing antibodies one month after the second dose of the vaccine was similar to that achieved in individuals aged 16–25 years, despite the reduced dose. The vaccine efficacy for the prevention of symptomatic disease was 90.7% (three children had COVID-19 in the experimental group versus 16 in the placebo group).

### 5.3. The Efficacy of the mRNA COVID-19 Vaccine among Children Aged ≥12 Years

According to the data presented in the first published clinical study of the Pfizer-BioNTech BNT162b2 vaccine [78] and post-marketing data following large-scale administration in Israel [79,80], the Pfizer-BioNTech BNT162b2 vaccine demonstrated ~95% efficacy in preventing symptomatic COVID-19 disease. Further research among adolescents aged 12–15 years demonstrated 100% efficacy for symptomatic illness; the antibody levels in this age group were higher than those of young adults aged 16–25 years [81].

A study was recently published by the CDC on adolescent COVID-19 vaccination [82], which was conducted during the Delta variant surge in the United States, between June 2021 and September 2021. A two-dose schedule of the Pfizer-BioNTech BNT162b2 vaccine was found to reduce the risk of COVID-19-related hospitalizations by 93% in adolescents aged 12–18 years. Additionally, among hospitalized adolescents, 97% were not vaccinated; among adolescents admitted to intensive care units or requiring mechanical ventilation or extracorporeal membrane oxygenation (ECMO), none were vaccinated against COVID-19. In view of the high efficacy of the vaccine in preventing severe complications of COVID-19 disease in adolescents, the CDC recommended vaccinating this age group against COVID-19 as early as possible.

### 5.4. The Effectiveness of the mRNA COVID-19 Vaccine in Preventing Transmission of Infection

In addition to the efficacy of the mRNA COVID-19 vaccine in preventing symptomatic disease, its efficacy in preventing asymptomatic infection was demonstrated in several studies [83,84,85,86]. These studies indicate the effectiveness of the vaccine also in reducing infectibility, even of the Delta variant. Real-world data from the United Kingdom of ongoing surveillance show a 2-to-3 times higher risk of asymptomatic or symptomatic infection with COVID-19 in unvaccinated compared to vaccinated persons. The data reflected the months in which Delta became the dominant COVID-19 variant, after adjusting for age and other variables [87].

The research institute of Clalit Health Services, the largest health maintenance organization in Israel [88], conducted a study of recently vaccinated adolescents aged 12–18 years. The two-dose schedule of the Pfizer-BioNTech BNT162b2 vaccine demonstrated ~93% effectiveness in preventing symptomatic disease and ~90% effectiveness in preventing COVID-19 infection caused by the Delta variant. Furthermore, in the Delta period, vaccinated persons were shown to be less contagious than unvaccinated persons, despite similar viral loads [89]. The efficacy of the vaccine against COVID-19 infectibility was shown to wane over time, thereby emphasizing the need for a booster dose of COVID-19 vaccines for long-term protection against further viral transmission. Correspondingly, a study from Singapore [90] (preprint, prior to peer review) demonstrated a shorter duration of COVID-19 transmission of the Delta variant in vaccinated than unvaccinated populations. 

In contrast to the above, a British study that examined household transmission in the Delta period showed similar infectibility between vaccinated and non-vaccinated persons [91]. However, secondary COVID-19 transmission from these breakthrough cases was lower than transmission from unvaccinated persons. The authors concluded that booster doses for COVID-19 vaccines should be given to reduce the transmission rates among households. Another British study that included both the Alpha and Delta variants [92] showed that the Delta variant is more contagious than the Alpha variant within households, but index cases that received two vaccine doses were less contagious than non-vaccinated persons. A Dutch study [93] undertaken during the Delta period (preprint, prior to peer review) also showed higher secondary transmission from unvaccinated than vaccinated persons. These three studies demonstrate that despite breakthrough infections within households, vaccination holds a protective effect in reducing secondary transmission from breakthrough cases. 

The CDC concluded that vaccinated people are significantly less contagious than non-vaccinated people and that their infectibility period is shorter. Hence, vaccinees help to reduce the transmission of COVID-19 because of their reduced chance of becoming infected and their shorter period of infection if they become infected. 

### 5.5. The Importance of Vaccination against Emerging SARS-CoV-2 Variants

Multiple SARS-CoV-2 variants circulating internationally raise constant global concern of the emergence of variants with increased transmissibility, increased virulence or decreased response to available vaccines. As global vaccine access is highly unbalanced, with high variability in vaccine coverage ranging from 1% to 70%, depending largely on a country’s wealth [94], SARS CoV-2 variants continue to emerge [4]. 

As of December 2021, the European Centre for Disease Prevention and Control (ECDC) [95] and the World Health Organization (WHO) [96] listed the following variants as variants of concern (VOCs): B.1.351 (Beta, first detected in South Africa), P.1 (Gamma, first detected in Brazil) and B.1.617.2 (Delta, first detected in India). The WHO also included the B.1.1.7 variant (Alpha, first detected in the UK). In November 2021, the ECDC and the WHO added the variant B.1.1.529, named Omicron, as a VOC [97]. The Omicron variant is a highly divergent variant with a large number of mutations, including 26–32 in the spike protein, some of which may be associated with immune escape potential and higher transmissibility. As of December 14, 2021, Omicron was identified in 77 countries [98]. Preliminary evidence from non-peer-reviewed studies and the considerably altered antigenic profile of the Omicron spike protein suggest reduced vaccine efficacy against infection and transmission [99,100,101].

Given the ongoing constraints of vaccine supply, some countries have extended the timing between vaccine doses in an effort to provide first doses to a greater number of people [102]. However, in response to emerging VOCs, some countries have reported reducing the previously extended timing between the first and second vaccine doses [103]. Furthermore, in response to the Omicron variant, several countries have reduced the time between the second and third COVID-19 vaccinations [104,105,106,107]. As of December 2021, most countries do not give boosters to children and adolescents. However, as the booster is considered to provide optimal protection against Omicron [108,109], a third dose, administered extensively in this population and even at a relatively short time following the second dose, may be deemed appropriate. Nevertheless, the rapid administration of full vaccination programs across all age groups, including children and adolescents, is critical to protect against emerging variants in the future. 

### 5.6. The Expected Benefit of Immunization in Reducing the Burden of COVID-19 Complications (Long COVID and MIS-C) in Children and Adolescents

Based on former knowledge regarding other vaccines’ performances in preventing viral diseases and their complications, COVID-19 vaccines are estimated to reduce the rate of late complications of COVID-19 disease, mainly by reducing infection rates. Additionally, a comprehensive study conducted in the United Kingdom provided supporting evidence for a decrease in disease complication rate [110]. Accordingly, the risk of long COVID-19 was reduced by 50% in vaccinated adults with breakthrough infection compared to unvaccinated infected adults. Protection against long COVID is probably even higher among vaccinees, as infection rates are lower among vaccinated than unvaccinated persons. 

### 5.7. COVID-19 Immunization and Prevention of MIS-C 

Case reports regarding MIS-C morbidity among fully vaccinated adolescents are rare. Of about 220 reported MIS-C cases in Israel to date, only one was an adolescent who received two doses of the COVID-19 vaccine ~4 months prior to MIS-C (unpublished data, personal communication). This supports the assumption that COVID-19 vaccination protects against the development of this complication. The CDC notes that immunization is expected to reduce disease complications, including long COVID and MIS-C syndrome, and cites this as a key consideration in favor of vaccinating children and adolescents [111]. The head of the CDC recommended [112] immunization against the SARS CoV-2 virus, inter alia, to reduce the risk of developing MIS-C.

An analysis conducted by the CDC [9] demonstrated that Pfizer vaccine given to children aged 5–11 years will prevent serious COVID-19 illness, including hospitalizations, intensive care unit admissions and MIS-C morbidity.

### 5.8. COVID-19 Vaccination of Recovered Children

During the discussion of the CDC Advisory Committee on Immunization Practices on 2 November 2021 [9], data from Pfizer’s Phase 3 study in children aged 5 to 11 years were presented. Nine percent of the study participants had positive blood serology for SARS CoV-2 at baseline. After two vaccine doses, the antibody levels of these seropositive children were higher than those of children with negative serology prior to vaccination. Importantly, children who had positive serology at the beginning of the study had fewer adverse events than their counterparts. In a follow-up of adolescents and adults who had positive serology prior to vaccination, no post-vaccination safety issues were encountered.

In conclusion, the CDC does not recommend serological testing prior to immunization [9] due to a lack of correlation between antibody level and protection rates. Furthermore, the benefits of vaccinating recovered children outweigh the disadvantages.

## 6. Vaccine Safety 

### 6.1. Safety of the mRNA COVID-19 Vaccine according to Research and Real-World Data

The original clinical study involving 40,000 individuals showed high safety of the Pfizer-BioNTech BNT162b2 COVID-19 vaccine [78]; the rate of significant adverse events occurring soon after vaccine administration was minimal. Further, following wide-scale administration in the population, a rare response of myocarditis was identified. 

In a follow-up study of 12–15-year-old adolescents [81], active surveillance for adverse events detected local side effects, mainly pain at the injection site, and systemic ones, mainly weakness, headache and chills. These adverse events were of mild-to-moderate severity and resolved within 24–48 h. No significant side effects were found in that clinical study. Based on those results, the regulatory approval for vaccination up to age 12 years has been extended by several regulatory authorities: in Canada, by the United States FDA [113], in the United Kingdom [114], and by the European Medicines Agency (EMA) [115]. After studying the efficacy and safety data and the benefits of vaccination against possible risks [116], the Vaccines and Related Biological Products Advisory Committee decided to recommend vaccination for ages 12 to 15 years. Data from one of the CDC safety monitoring systems, Vaccine Adverse Event Reporting System, showed that the rate and nature of both adverse events and severe adverse events among 12–15-year-old adolescents, who received about six million vaccine doses in total, was similar to that observed among adolescents and young adults aged 16–25 years [117].

Data as of 25 October 2021 that were retrieved from IMoH vaccine safety monitoring, including administration of 6,223,401 first doses, 5,719,151 second doses, and 3,916,431 third doses, demonstrated that the vast majority of the vaccine-related side effects were mild and transient. 

### 6.2. Safety of the mRNA COVID-19 Vaccine among Children Aged 5–11 Years

Prior to the FDA discussion on the approval of the Pfizer-BioNTech BNT162b2 COVID-19 vaccine for ages 5–11 years, which was held on 26 October 2021, data from the clinical study were published for this age group [77]. For the safety clinical trial, 2268 children were initially recruited: 1518 for the vaccine group and 750 for the control group. A subsequent group of 2379 children was later recruited at the request of the FDA. The safety data showed favorable results. The reported adverse events were for the most part local and transient: local pain at the vaccine site (71%), fatigue (39%) and headache (28%). Reactions were more frequent after the second than the first dose of the vaccine and resolved within one or two days. The incidence of adverse events was low relative to that observed in 16–25-year-old adolescents and young adults. The incidence of adverse events in the subsequent cohort was similar to that of the first cohort. Of the 3109 children vaccinated, four had serious adverse events in the vicinity of receiving the vaccine—all the events were recognized by the FDA as non-vaccine-related reactions. There were no reports of peri/myocarditis (it should be noted that the number of participants was too small to detect a rare phenomenon such as myocarditis after vaccination) or anaphylaxis; there was no mortality. As of December 2021, the Pfizer-BioNTech BNT162b2 COVID-19 vaccine is the only vaccine approved for the 5–11-year age group. The use of the Moderna mRNA 1273 COVID-19 vaccine (Spikevax) for this age group is currently being assessed by the EMA. Trials in children as young as age 3 years were completed for two inactivated vaccines (Sinovac-CoronaVac and BBIBP-CorV), and these products were approved by Chinese authorities for the age indication of 3–17 years. Though these vaccine products have received emergency use listing for adults, they have not yet received this approval by the WHO for children. Several other COVID-19 vaccines are undergoing trials in younger age groups (including as young as age 6 months), but results have not been published [118]. 

In accordance with FDA approval of the Pfizer-BioNTech COVID-19 vaccine for children aged 5–11 years, on 25 November 2021, the EMA issued a positive opinion for its use in children aged 5–11 years, as did the National Advisory Committee on Immunization (NACI) of the Government of Canada [119].

In addition to the above, according to the December 2021 report of the ECDC, several countries are planning to expand vaccination to children under age 12 years (i.e., Czechia, Lithuania, Hungary) and others report that this subject is under discussion (i.e., Belgium, Croatia, Latvia, Luxembourg, Malta, Netherlands, Poland, Portugal, Spain) [120].

Additional safety data are available from monitoring a cohort of Israeli children aged 5 to 11 years with severe co-morbidities who were vaccinated prior to the regulatory approval as a “companionate use”, off-label prophylaxis. From 1 August 2021 to 24 October 2021, 163 children were vaccinated with a first dose and 128 children with a second dose. As of 24 October 2021, only five reactions were reported adjacent to vaccine receipt: four mild reactions (fever, pain, weakness) and one severe adverse event of generalized seizure in an 8-year-old child.

As of late November, more than 3.6 million children in the United States received one dose and 135,000 received two doses [16]; to date, no exceptional safety reports have been recorded.

The biological likelihood that a completely new serious complication of vaccination, not yet known, with a clear excess risk in children aged 5 to 11 years, is minimal, but cannot be completely ruled out. The potential benefits and risks of COVID-19 vaccination in children are summarized in Table 1.

### 6.3. Myocarditis following COVID-19 Vaccination

According to IMoH data, myocarditis following vaccination occurred with a maximum incidence of 1:6637 in young vaccinated males aged 16–19 years soon after receiving the second dose [121,122]. In other age groups, including younger adolescents aged 12 to 15 years, the phenomenon was found to be less common, as well as after receiving a third dose of the vaccine [3].

The CDC detected an over-prevalence of peri/myocarditis in 12-to-39-year-old adolescents and adults at a rate of ~1:100,000, with the highest prevalence occurring after the second vaccine dose in adolescent boys, aged 12 to 17 years, with an incidence of about 1:16,000 vaccinees [123,124], similar to the observation in Israel. 

A US study [125] (prior to peer review) found that among males over age 12 years, the risk of developing myocarditis as a result of contracting the virus was six times higher than the risk of developing this phenomenon following immunization. For males at the age of maximum risk, the rate of myocarditis, as a result of infection with the virus, was approximately 1:1600 SARS-CoV-2-positive cases.

A large-scale study conducted in Israel by Clalit Health Services [126] that included all age groups demonstrated an excess risk of myocarditis post-mRNA COVID-19 vaccination compared to the general population. Nevertheless, COVID-19 infection was associated with a greater risk of myocarditis. Other serious conditions that were associated at a higher rate with COVID-19 infection than following the vaccination included hypercoagulability (deep vein thrombosis and pulmonary emboli), acute myocardial infarction, cerebral hemorrhage, arrhythmias and acute kidney injury. In concert with these findings, another Israeli study from Clalit Health Services [127] showed that among males aged 16–39 years, mRNA COVID-19 vaccination was associated with an increased risk of myocarditis; however, COVID-19 infection was associated with a higher risk of myocarditis in all age groups and in both genders.

The incidence of myocarditis post-vaccination is expected to be lower at age 5 to 11 years than in older males for several reasons. First, according to the literature, during the pre-COVID era, the overall incidence of myocarditis in the 5-to-11-year age group was lower than in males aged ≥16 years [128]. Second, a lower incidence of myocarditis was observed in the 12-to-15-year age group compared to older adolescents. Third, according to experts, as reflected in the discussion of the FDA expert committees held on 26 October 2021, and in the discussion of the Advisory Committee on Immunization Practices on 2 January 2021 [9], a lower incidence of myocarditis may be expected in this age group due to the reduced dose (10 mcg instead of 30 mcg) and the low propensity to develop myocarditis for this age group. 

According to a series of scientific publications, myocarditis post vaccination has a relatively benign and mild clinical course compared to SARS-CoV-2 infection-related myocarditis: A study that followed young people who developed vaccine-related myocarditis in Israel [129] found that the vast majority had mild myocarditis. Moreover, among all those with post-discharge follow-up, no echocardiographic evidence of residual cardiac damage was found. According to the CDC [130], vaccine-related myocarditis in young people is usually mild and is characterized by rapid recovery for the vast majority. Recovery from vaccine-related myocarditis is much faster than the course of recovery described in “classic” myocarditis and is characterized by rapid regression of symptoms and rapid recovery of cardiac function [131]. Thus, these findings further divert the risk–benefit balance of immunization in children and adolescents, as even myocarditis, the most severe known risk following immunization with mRNA vaccines, carries a benign clinical course in the vast majority of cases.

In conclusion, the risk of myocarditis, and as a result, long-term sequelae, is higher in relation to SARS-CoV-2 infection than to the mRNA vaccine among all age groups investigated, i.e., ages over 12 years. Furthermore, the data suggest that myocarditis due to infection with the virus, caused by both serious COVID-19 and MIS-C syndrome, is more dangerous than that caused by mRNA vaccination. This should be considered together with the assumption of widespread COVID-19 infection in the population and the recognition that myocarditis is the most significant side effect of vaccination in children and adolescents. Thus, immunization is likely to be of significant benefit in reducing the incidence of myocarditis and sequalae caused in the long term.

The risk of severe myocarditis following vaccination is significantly lower than the risk of developing MIS-C syndrome due to infection for several reasons. MIS-C syndrome, which is the most serious complication of SARS-CoV-2 infection in children and adolescents, is often characterized by a life-threatening clinical course, while the most dangerous complication of the mRNA vaccine in this age group is fulminant myocarditis. In an Israeli study conducted among Clalit Health Services insurees [129], the incidence of severe myocarditis following vaccination was estimated as 1:300,000 among vaccinated males aged 16–29 years (0.34 per 100,000). As mentioned, in children and adolescents younger than age 16 years, the incidence of vaccine-related myocarditis is lower, including fulminant myocarditis. In contrast, the prevalence of MIS-C syndrome is approximately 1:3000 in children and adolescents with a proven COVID-19 infection. In this context, even among adolescents in the age group with the highest rate of vaccine-related myocarditis side effects, the risk of the most serious complication following infection is two orders of magnitude higher than the most severe complication following mRNA vaccination. This gap increases for girls and other age groups.

The risk of developing a serious illness following SARS-CoV-2 infection and its complications is significantly higher than the risk of vaccine-related myocarditis vaccination at any age. This was demonstrated by a model presented at the FDA discussion [132], which assessed the risk from the vaccine, i.e., morbidity and admissions due to myocarditis, versus the benefit of vaccination in children aged 5 to 11 years. The benefit was reflected by the prevention of serious morbidity and hospitalizations due to virus infection. Six scenarios were selected, which differed in their fundamental assumptions regarding the extent of morbidity, the benefit of vaccines and the prevalence of myocarditis following vaccination in 5-to-11-year-old children. For each million vaccinated, the model calculated the number of severe infections (hospitalizations and intensive care) and the number of deaths due to COVID-19 that could be avoided compared to those caused by vaccine-related myocarditis over 6 months. The benefit from the vaccine was found to clearly outweigh its potential risk, even in low-morbidity scenarios given the lower detriment of vaccine-dependent myocarditis versus coronary morbidity.

The above model estimated that the incidence of vaccine-related myocarditis in 5-to-11-year-old children would be similar to that found in 12-to-15-year-old children. However, if the incidence among the younger children is actually lower (due to their younger age and reduced vaccine dose), as was estimated, the risk–benefit ratio is even more “lucrative” in favor of immunization for this age group. Notably, the model only assessed the expected morbidity among children at these ages and did not weigh other benefits of vaccination, such as reducing the probability of transmitting the infection to others [132]. According to a CDC model [111] at any age over 12 years, including adolescents aged 12–17, the volume of SARS-CoV-2 morbidity-related hospitalizations that would be prevented by vaccination is higher than the number of vaccine-related cases of myocarditis per million mRNA vaccine doses.

An analysis conducted in the IMoH Department of Epidemiology showed that even in Israel, at all ages and in both genders, the risk of developing a serious disease due to infection with the virus is higher than the risk of developing myocarditis due to immunization [3].

Summarizing the available data, it seems that for all age groups, including young males, the risk of developing a serious illness due to SARS-CoV-2 infection and its complications is substantially higher than the risk of developing myocarditis following vaccination. The total benefit conferred by reducing children and adolescents to SARS-CoV-2 morbidity by means of vaccination can be compared with the risk of developing myocarditis following mRNA vaccines. In Israel, the calculated risk of SARS-CoV-2-related hospitalization in children is 1:200 and hospitalization due to a moderate/severe/critical disease or its complications, including MIS-C syndrome, is about 1:825 confirmed cases. While the risk of developing long COVID is still debatable, it is at least one percent of proven cases of COVID-19. The possibility of long-term complications involving infection, as with other viruses, should also be considered. These risks should be weighed against the maximal incidence of vaccine-related myocarditis, which was found to be about 1:6600 among young males aged 16–19 years; the vast majority of those affected experienced a benign clinical course and rapid recovery.

We presume that the incidence of vaccine-related myocarditis among children aged 5 to 11 years will be equal to or even lower than that observed in older males. This, together with the assumption that no completely new serious side effect is likely to emerge in this age group, yields an expected improved risk–benefit balance relative to older boys in favor of vaccination.

### 6.4. COVID-19 Vaccines Show No Signs of Harming Female or Male Fertility

Female fertility—Several published studies reported no evidence that the various COVID-19 vaccines affect a woman’s fertility in all its aspects. These include no damage to the ovarian reserve and ovarian function [133,134], and no damage to the implantation of the fetus in the uterus and the ability of the endometrium to maintain pregnancy [135,136,137]. The vaccine was not shown to increase incidences of miscarriage or preterm birth [137,138]. A large Israeli study (prior to peer review) [139] demonstrated no harm to female fertility.

Male fertility—No evidence of impaired male fertility has been shown. Studies conducted in the United States [140] and in Israel [141] (prior to peer review) demonstrated no effect of mRNA vaccines on sperm parameters and no effect on male fertility.

### 6.5. Lack of Correlation between Increased Menstrual Bleeding and Female Infertility 

A recently published review [142] noted the reporting of bleeding irregularity after various types of vaccinations, including mRNA vaccines and adenovirus vector vaccines, as well as non-corona vaccines, such as those for influenza and human papillomavirus. The menstrual cycle returns to normal in the subsequent cycles. The authors emphasized that there was no evidence that vaccination harms woman’s fertility. Further, the British Association of Gynecologists stated [143] that even if a change in menstruation occurs, usually in the subsequent cycle, regularity returns, and that no evidence exists that these changes impair fertility or the ability to conceive and give birth.

Both the EMA [144] and the British National Medicines Agency [145] stated that no causal relationship has been established between vaccination and menstrual bleeding irregularity.

## 7. Summary and Recommendations

Most children who become infected with SARS-CoV-2 are asymptomatic or have mild symptoms, and the vast majority recover without sequelae. In general, COVID-19 morbidity and mortality are significantly lower in children than in adults; however, the risk of severe COVID-19 in children is not negligible, even among children without background illness. 

In Israel, by October 2021, 398 children up to age 18 years were hospitalized for moderate/severe/critical illness from COVID-19, and at least 220 children were hospitalized for MIS-C syndrome. Therefore, in this region, the risk of hospitalization due to significant morbidity is about 1 in 825 children with a proven SARS-CoV-2 infection. The prevalence of long COVID is yet to be determined; however, it evidently presents in at least 1% of proven COVID-19 cases, and may substantially impair a child’s quality of life.

A controlled clinical study found Pfizer’s mRNA COVID-19 vaccine to be 91% effective in preventing symptomatic disease in children aged 5 to 11 years.

In large-scale studies, the vaccine was found to have an excellent safety profile for children aged 12 years and older. Similarly, the Pfizer study showed an excellent safety profile for children aged 5 to 11 years.

At present, the most significant adverse event for which mRNA vaccines confer excess risk in the general population, and in young people in particular, is vaccine-related myocarditis. This is assumed to apply also to children aged 5 to 11 years. The incidence of vaccine-related myocarditis in this age group is expected to be equal to or less than that of males aged over 12 years. The incidence of myocarditis post vaccination was been shown to be highest among adolescent males aged 16 to 19 years at a rate of about 1:6600. About 95% of vaccine-related myocarditis events have a mild and transient clinical course. Vaccine-related fulminant myocarditis is extremely rare.

A series of studies did not find an association between fertility impairment and mRNA vaccines. 

Children are also highly susceptible to downstream effects of COVID-19, including social isolation and interruption in education. Closing the education system entails substantial psychosocial harm to children and adolescents; thus, maintaining school routine is of immense value to their mental and physical health, and to the completion of learning gaps of the past year.

Children have an important role in the transmission of SARS-CoV-2. The last months of the COVID-19 pandemic in Israel have witnessed an increasing proportion of transmissions by children to both other children and adults.

Prevention is central to the field of pediatrics. Children have been vaccinated routinely against preventable diseases for many years, not just during an epidemic. The availability of the COVID-19 vaccine reduces the risks of COVID-19.

Following booster vaccinations to the adult population in Israel [146] COVID-19 morbidity decreased during September–October 2021 [1]. However, during November 2021, moderation and even reversal of this trend were observed in face of the morbidity wave in Europe and the United States. New variants are expected to emerge, and the disease breaks out particularly in populations with low immune coverage, the main one being children. 

In addition to the above, the emergence of a highly contagious Omicron variant further underscores the importance of rapid immunization of children, as well as the general population. Unvaccinated persons are currently defenseless and should be given every protection possible. Concern has arisen that the Omicron variant may reduce the usefulness of the vaccine by evading antibody protection. Nonetheless, some and even significant protection from the vaccine is expected due to the presence of other components in the immune system, such as T cells, which vary less between variants. An Omicron-specific vaccine may become available in only a few months. In the meantime, children and other unvaccinated persons will be exposed to the disease, both from the Omicron and Delta variants, while having no protection.

The entire population, or at least the vast majority, may likely become exposed to SARS-CoV-2 at some point. In light of the predicted trends of the epidemic, the virus may become endemic and most of humanity, including children, may eventually be exposed to it. Therefore, the alternatives will be exposure to the virus as a vaccinated or an unvaccinated person.

In conclusion, after an in-depth examination of the literature and the available data on the burden of COVID-19 disease in children and on childhood vaccination against COVID-19, we inferred that the benefits of prevention of COVID-19 disease via vaccination outweigh the possible risks of vaccination. Childhood vaccination against COVID-19 protects against the direct acute and long-term effects of COVID-19 disease. It bears the potential benefit of protection against COVID-19-associated hospitalization occurring at a rate of 1:200, protection against a moderate-to-critical condition of acute COVID-19 disease occurring at a rate of 1:900, protection against COVID-19-associated myocarditis occurring at a rate of 1:1600, protection against MIS-C occurring at a rate of 1:3000 and protection against long COVID occurring at a rate of ~1:100. In addition, vaccination protects from indirect effects of the COVID-19 disease, such as school closure, community transmission and the emergence of viral variants. The risks of COVID-19 vaccination in children are mainly confined to the risk of vaccine-associated myocarditis, which is estimated at 1:6000 and is usually mild. 

Therefore, we at the Israeli Association of Pediatricians and the Israeli Society for Pediatric Infectious Diseases recommend the use of mRNA COVID-19 vaccines for children aged 5 years and older while providing reliable and valid information that is accessible and transparent to parents, adolescents and children (Table 2).

## 8. Recommendations

1. Vaccination against COVID-19 is recommended for children aged 5 years or older while providing full information to the parents, children and vaccinated adolescents regarding the effectiveness of the vaccine, the importance of disease prevention and the safety of the vaccine.
2. Development of anaphylaxis or myocarditis following mRNA COVID-19 vaccination should serve as a contraindication for additional doses of the vaccine.
3. A comprehensive vaccine safety follow-up program will be conducted by the Ministry of Health in Israel.

## Figures and Tables

**Table 1:** Summary of benefits and risks of vaccination of children against COVID-19.

Potential Benefits and Risks of Childhood Vaccination against COVID-19		
Potential Benefits	Potential Risks	
Prevention of COVID-19-associated hospitalization	Local transient vaccine-related side effects	7:10
Protection against a moderate-to-critical condition of COVID-19 disease	Vaccine-associated myocarditis	1:6600 *, mild in 95%
Protection against COVID-19-associated myocarditis	Vaccine-associated severe myocarditis	1:300,000 **
Protection against MIS-C	Transient menstrual bleeding irregularity	
Prevention of MIS-C-associated death		
Protection against long COVID		
Prevention of possible unknown long-term effects		
Avoidance of isolation and school closure		
Reducing community transmission		
Reducing emergence of VOCs		

MIS-C—multisystem inflammatory syndrome in children; VOCs—variants of concern. * Maximal estimated risk among males aged 16–19 years. ** Maximal estimated risk among males aged 16–29 years.

**Table 2:** Recommendations of the Israeli Association of Pediatricians and the Israeli Society for Pediatric Infectious Diseases regarding vaccination of children against COVID-19.

We recommend the use of the Pfizer-BioNTech COVID-19 vaccine for children aged 5 years and older while providing accessible and transparent information to the parents, adolescents and children regarding the effectiveness of the vaccine, the importance of COVID-19 prevention and the safety of the vaccine.
The second dose of the Pfizer-BioNTech COVID-19 vaccination series should be deferred in children who experience anaphylaxis or myocarditis following the first dose of the Pfizer-BioNTech COVID-19 vaccine.

## Data Availability

Not applicable.

## Abbreviations

COVID-19—coronavirus disease 2019; SARS-CoV-2—severe acute respiratory syndrome coronavirus 2; MIS-C—multisystem inflammatory syndrome in children; IMoH—Israeli Ministry of Health; CDC—Center for Disease Control and Prevention; FDA—Food and Drug Administration; mRNA—messenger ribonucleic acid; EMA—European Medicines Agency; VOCs—variants of concern; ECDC—European Centre for Disease Prevention and Control; WHO—World Health Organization.
